# Effect of honey bee venom on the histological changes of testes and hormonal disturbance in diabetic mice

**DOI:** 10.14202/vetworld.2022.2357-2364

**Published:** 2022-09-30

**Authors:** Sattar J. J. AL-Shaeli, Talal Jabal Hussen, Ali M. Ethaeb

**Affiliations:** 1 Department of Medical Basic Sciences, College of Dentistry, Wasit University, Wasit, Iraq; 2 Department of Anatomy and Histology, College of Veterinary Medicine, Wasit University, Wasit, Iraq

**Keywords:** diabetic mice, hyperglycemia, Sertoli cells, spermatogonia

## Abstract

**Background and Aim:**

Hyperglycemia associated with hyper- or hypo-insulinemia is a hallmark of type 2 diabetes mellitus, which is firmly linked to decreased male infertility. Recently, bee venom (BV) has shown potential health prosperities, including antidiabetic; however, no study focuses on the effect of BV on male fertility in diabetic conditions. This study aimed to detect the effect of BV on histological and hormonal alteration of the testis in diabetic mice.

**Materials and Methods:**

Twenty adult male mice were selected and assigned to four groups: Control, diabetic (150 mg/kg alloxan), BV1 (diabetic + 0.5 mg/kg BV), and BV2 (diabetic + 1 mg/kg BV). After 35 days, the serum levels of glucose, insulin, testosterone, follicular-stimulating hormone, luteinizing hormone, and prolactin were estimated. The histological structure of the testes was also evaluated.

**Results:**

Alloxan-induced hyperglycemia and decreased insulin concentrations were reversed significantly by BV. Furthermore, diabetic mice exhibited various alterations in fertility hormones, while these disturbances were improved considerably to normal concentrations by BV. Similarly, alloxan-induced changes in sperm and testis histological parameters such as motility, viability, abnormality, sperm count, the number and diameter of seminiferous tubules, and the number of Leydig and Sertoli cells were significantly ameliorated to the normal condition by BV. Changes in the number, size, and shape of seminiferous tubules, the number of Leydig and Sertoli cells, and initial degeneration and vacuolization in interstitial cells and spermatogonia and spermatocyte were seen in diabetic mice. All these changes were shifted almost to normal structure by BV.

**Conclusion:**

The BV could be used as an alternative therapeutic agent that manages the markers related to diabetic conditions concomitant with the improved histological structure of the testes and hormone production to accelerate male fertility.

## Introduction

Chronic deregulation of glucose and lipid metabolism is a consequence of the impaired and insufficient secretion of insulin associated with beta-cell failure, which are the main remarks of type 2 diabetes mellitus (T2DM) [1, 2]. The latter is a multifactorial metabolic disease, leading to hyperglycemia and hyperlipidemia, hyper- or hypo-insulinemia [3]. These factors contributed to reactive oxygen species elevating because of mitochondrial dysfunction and impairment of the antioxidant system [4]. Accordingly, oxidative stress is initiated with the generation of inflammatory mediators, causing low to moderate systemic inflammation as a characteristic hallmark of T2DM [5]. The incidence of T2DM is elevated each year with the rise of global concern as the world’s elderly population grows dramatically with no effective prevention and management strategies for diabetes [6]. Despite the worldwide effort and high expenditure, the disease prevalence is still increasing and will reach nearly 800 million cases by 2045 [7]. Therefore, it is considered an endemic metabolic alteration worldwide.

Male infertility is a comorbidity related to T2DM [8]. In addition to diabetes, several chronic diseases, obesity, and lifestyle, such as the type of diet, sedentary, smoking, environmental factors, stress, sleeplessness, psychological factor, etc., could involve male infertility [9]. Infertility is a global well-being issue that occurs when the couple is not conceived after one year of regular unprotected intimacy [10]. Around 8–12% of couples become infertile, and the male factor represents half of all infertility cases [11]. Global male infertility is rising dramatically to 768/100,000 in 2017 compared to females 1571/100,000; thus, the male factor is responsible for nearly 33% of all global infertility [12]. Male infertility is parallel increases with the rising trend in the incidence of T2DM.

Moreover, T2DM contributes to testicular deterioration resulting in male infertility [13]. Although the mechanism of T2DM-induced male infertility is not fully understood, continuous hyperglycemia increases the generation of ROS. It promotes oxidative stress associated with a declining antioxidant system to scavenge free radicals [14]. All these markers triggered the testicular tissue and endocrine system, which caused testicular and hormonal dysfunction, leading to male infertility [15].

Bee venom (BV) is used in ancient alternative apitherapy to prevent, control, and cure several diseases [16]. The BV exhibits several bioactive constituents, including melittin, apamin, phospholipase A2-induced mast cell degranulating, hyaluronidase, acid phosphomonoesterase, and other compounds that possess various cellular actions [17]. Therefore, the health properties and potential benefits are attributed to these bioactive compounds. These health properties are anti-cancer [18], antiviral [19], wound healing [20], anti-inflammatory, and antioxidant [21, 22]. Globally, limited studies identified the potential effect of BV by assessing the changes in the diabetic markers and ameliorating pancreas and liver histology [22, 23]. Some studies determine the consequences of diabetes on the histological and hormonal changes in the testes [24]. However, no study identifies the role of BV on testicular histology and hormonal changes in mice with T2DM.

Thus, this study aimed to detect the antidiabetic effect and role of BV in improving testicular histological structure and level of hormones in diabetic mice.

## Materials and Methods

### Ethical approval

The present study was mediated in accordance with the regulation of the Scientific Committee/College of Dentistry, University of Wasit, Wasit Province, Iraq, and the work was approved under license no. 392-12/4/2022.

### Study period and location

The study was conducted from April to May 2022 in Scientific Laboratory and Animal House of AL-Nahreen University (Baghdad, Iraq).

### Induced T2DM in laboratory animals

Adult 20 male laboratory mice (*Mus musculus*), 50–60 days old and weighing about 25–36 g, were purchased from the Ministry of Science and Technology animal house. The mice were placed in separate cages, transported to a new place in the AL-Nahreen Animal House (Baghdad, Iraq), and left for one week with free access to food and water for acclimation under standard conditions with 12:12 light and dark cycle. Fifteen mice were subjected to overnight fasting, and then 150 mg/kg of ready alloxan (Sigma-Aldrich, UK) was injected intraperitoneally (IP). The mice were then provided with high glucose-free water for 3 days, and on the 4^th^ day, the level of circulatory blood glucose was estimated using the blood drop from the tail. The mice with ≤200 mg/dl circulatory blood glucose were considered non-diabetic and subjected to another dose of alloxan at 150 mg/kg following the same previous protocol. After 4 days of the second attempt injection of alloxan, the induced diabetes was confirmed in all mice as the glucose level was more than 200 mg/dl.

### Preparation of BV

The BV was obtained from the local market (Wasit Province, Iraq) and was dissolved in an accountable amount of doubled distilled water. The BV was kept at −20°C until needed.

### Experimental study design

In this study, the mice were categorized into four groups as follows:

Control (Group-I): Contained five mice neither diabetic nor treated with BV and only received food and water.

Diabetic (Group-II): Contained five diabetic mice that were not treated with any concentration of BV.

Low BV (Group-III): Contained five diabetic mice which IP injected daily with a single dose of 0.5 mg/kg of BV for 35 days.

High BV (Group-IV): Contained five diabetic mice which IP injected daily with a single dose of 1 mg/kg of BV for 35 days.

On day 35, mice were immediately injected with 0.3 mg/kg ketamine and 0.1 mg/kg lidocaine. The blood was directly collected from the heart, and the serum was obtained by centrifuging at 1000× *g* for 15 min. Then, the serum was kept at −20°C until analysis. Furthermore, the mice testes were dissected and washed with distilled water, and dry it before being transferred into 10% formalin for 48–72 h.

### Serological examination

Sandwich ELISA kits were used to determine the glucose concentration (AGAPPE, India), insulin level (Monobind Inc., USA), testosterone hormone (Monobind Inc.), luteinizing hormone (LH) (Monobind Inc.), follicular-stimulating hormone (FSH) (Monobind Inc.), and prolactin hormone (Monobind Inc.) in serum samples, according to the manufacturer protocol.

### Histological examination

Formalin-fixed testes were washed with 70% alcohol and proceeded for ordinary histological examination. The histological sections were stained with hematoxylin and eosin. The histological slides were evaluated under the light microscope (Olympus, Japan), and the images were processed and captured using a camera (Optica Italy). The diameter of the seminiferous tubule was measured by the mitotic program.

### Sperm examination

The caudal epididymis of the mice was dissected and placed into 2 mL phosphate buffer saline in a Petri dish. Eosin stain of about three drops was placed onto the new histological slide and diluted semen of about one drop was added to the stain and mixed gently. The semen was spread on the slide thoroughly and dried. Then, the slide was examined under the microscope.

### Statistical analysis

The data were processed by Microsoft Office Excel version 2019 (Microsoft, USA) and analyzed by GraphPad Prism version 6 (GraphPad Software Inc., USA). One-way analysis of variance with Turkey’s *post hoc* was used for multiple comparisons. The significant p values were presented as <0.05 (*), <0.01 (**), <0.001 (***), and <0.0001 (****) when comparing between groups. The continuous data were presented as mean ± standard error of the mean.

## Results

### Bee venom reduced blood glucose levels and elevated insulin concentration in diabetic mice

**Figure-1:**
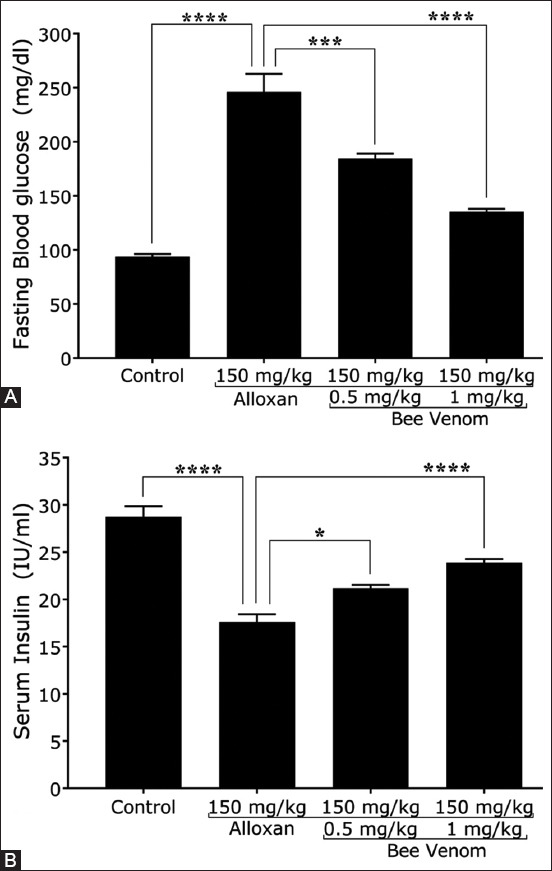
Bee venom (BV) ameliorates the levels of glucose and insulin in diabetic mice. (A) BV significantly decreased the glucose concentration in diabetic mice. (B) BV significantly elevated the insulin concentration in diabetic mice, n = 5.

The mice with alloxan-induced diabetes showed a significant elevation of the blood glucose and decreased insulin concentrations by 162% ± 6.8% and 38.8% ± 4.6%, respectively. These changes were significantly improved in mice injected with BV. The blood glucose level was notably reduced by 25% ± 2.5% and 45% ± 2% in mice that received 0.5 mg/kg and 1 mg/kg of BV, respectively (Figure-1A). Similarly, significant increases in insulin levels by 20.2% ± 1.7% and 35.6% ± 1.5% were observed in mice treated with 0.5 mg/kg and 1 mg/kg of BV, respectively (Figure-1B).

### Bee venom enhances the disturbance of male fertility hormones in diabetic mice

**Figure-2:**
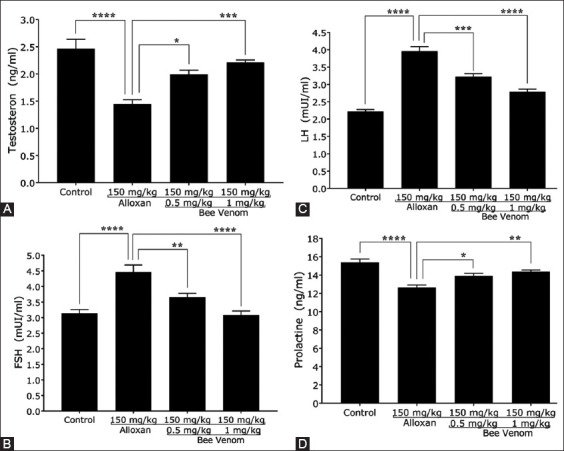
Bee venom (BV) ameliorates the level of fertility hormones in diabetic mice. (A) BV significantly elevated the testosterone concentration in diabetic mice. (B) BV significantly decreased the follicular-stimulating hormone concentration in diabetic mice. (C) BV significantly decreased the luteinizing hormone concentration in diabetic mice. (D) BV significantly elevated the prolactin concentration in diabetic mice, n = 5.

The levels of male fertility hormones, including testosterone, FSH, LH, and prolactin, were measured in all experimental mice. A significant decline in the mean concentration of testosterone and prolactin hormones was observed by 41.25% ± 5.4% and 17.9% ± 2.1%, respectively, in diabetic mice. These changes in two hormones were significantly reversed by 37.5% ± 4% and 52.5% ± 2.1% in mice with 0.5 mg/kg dose of BV, and 10% ± 2% and 13.75% ± 1.2% in 1 mg/kg dose of BV, respectively (Figures-2A and D). Furthermore, diabetic mice showed elevated levels of FSH and LH, with the mean concentration of FSH of 42.2% ± 5% and that of LH of 78.7% ± 3.2%. While diabetic mice injected with 0.5 mg/kg and 1 mg/kg dose of BV showed a significant reduction of the mean concentration of FSH by 18.1% ± 3.4% and 30.9% ± 4.1% and remarkable reduction of the mean concentration of LH by 18.6% ± 2.7% and 29.6% ± 2.6%, respectively (Figures-2B and C).

### Bee venom ameliorated the disturbance of sperm characteristics in diabetic mice

**Figure-3:**
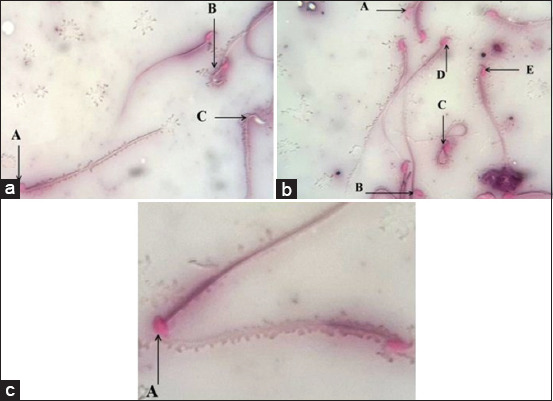
Morphological detection of the sperms in the study mice. (a) A. Dead sperm. B. Abnormal sperm. C. Life sperm. (b) A. Life sperm. B. Dead sperm. C. Abnormal sperm. D. Polygonal head sperm. E. Pyriform head sperm. (c) A. Hammer head sperm.

The sperm characteristics, including sperm count and the proportion of sperm motility, dead, and abnormality, were identified by a light microscope. The sperm motility was significantly decreased by 55% ± 4.1% in diabetic mice. In contrast, the low and high doses of BV restored the motility by 33.8% ± 3.1% and 86.3% ± 2.3%, respectively (Table-1). The proportion of dead sperm in diabetic mice was notably increased by 71.6% ± 6%. This high percentage of dead sperm was significantly enhanced by 21.1% ± 4.4% and 36.6% ± 4.8% in response to BV, respectively (Table-1). Administration of low and high doses of BV caused significant enhancement of sperm count up to 21% ± 5% and 34.4% ± 5.3%, respectively (Table-1). Furthermore, sperm abnormality was increased by 117.5% ± 5.7% in diabetic mice, and this was significantly reduced by 23.6% ± 4.8% and 40% ± 4.8% in response to low and high doses of BV, respectively (Table-1 and Figure-3).

**Table-1:** Bee venom ameliorates sperm parameters in diabetic mice.

Parameters (mean ± SEM)	Groups			

	Control	Diabetic	Diabetic+0.5 mg/kg BV	Diabetic+1 mg/kg BV
Motility %	90.8 ± 2.1	40.8 ± .7 p , 0.0001, ****	54.6 ± 1.7 p = 0.0003, ***	76 ± 1.8 p , 0.0001, ****
Dead %	20.4 ± 1.1	35 ± 2.1 p , 0.0001, ****	27.6 ± 1.2 p = 0.0106, *	22.2 ± 1.1 p , 0.0001, ****
Abnormalities %	16 ± 0.7	34.8 ± 2 p , 0.0001, ****	26.6 ± 1.3 p = 0.0026, **	21.6 ± 1 p , 0.0001, ****
Count×10^7^	21.4 ± 1.2	39 ± 1.5 p , 0.0001, ****	30.8 ± 1.6 p , 0.0043, **	25.6 ± 1.4 p , 0.0001, ****

The diabetic mice were injected with 0.5 and 1 mg/kg with BV for 35 days. The specific sperm parameters were measured. The alloxan significantly induced reduction of sperm motility with the elevation of percent of dead sperm, abnormality, and count. Whereas BV significantly improved these parameters to nearly normal, n = 5. BV=Bee venom, SEM=Standard error of the mean

### Bee venom improves the disturbance of testis histological parameters in diabetic mice

The histological features of the testis, including the number and diameter of the seminiferous tubules and the number of Leydig and Sertoli cells, were determined. The alloxan significantly reduced the above characters by 51% ± 3.8%, 34.7% ± 1.7%, 47% ± 6.4%, and 38.3% ± 3.5%, respectively. These reductions in all histological parameters of the testis were remarkably enhanced in response to the low and high doses of BV. The significant increases in the low and high doses of BV were 34.6% ± 3.3% and 47.4% ± 5.3% in the number of seminiferous tubules, 13.3% ± 1.2% and 25.2% ± 1.2% in the diameter of seminiferous tubules, 30.7% ± 3.2% and 54.9% ± 2.5% in the number of Leydig cells, and 20% ± 3% and 47% ± 3.7% in the number of Sertoli cells, respectively (Table-2).

**Table-2:** Bee venom ameliorates the testes histological parameters in diabetic mice.

Parameters (mean ± SEM)	Groups			

	Control	Diabetic	Diabetic+0.5 mg/kg bee venom	Diabetic+1 mg/kg bee venom
Seminiferous tubules (number/field (10×)	31.8 ± 1.4	15.6 ± 0.6 p , 0.0001, ****	21 ± 0.7 p = 0.0098, **	23 ± 1.2 p , 0.0006, ****
Seminiferous tubules (mm/field)	232.2 ± 2.3	211 ± 3.5 p , 0.0001, ****	239 ± 2.8 p , 0.0001, ****	264.2 ± 3.2 p , 0.0001, ****
Leydig cells number	57.8 ± 1.5	30.6 ± 2 p , 0.0001, ****	40 ± 1.3 p = 0.0025, **	47.4 ± 1.2 p , 0.0001, ****
Sertoli cells number	32.4 ± 1	20 ± 0.7 p , 0.0001, ****	24 ± 0.7 p = 0.028, *	29.4 ± 1.1 p , 0.0001, ****

The diabetic mice were injected with 0.5 and 1 mg/kg with BV for 35 days. The specific histological parameters were measured. The alloxan significantly induced a reduction in the number and diameter of seminiferous tubules, number of leydig and sertoli cells. Whereas BV significantly improved these parameters to nearly normal, n = 5. BV=Bee venom, SEM=Standard error of the mean

### Bee venom ameliorated the histological deterioration of the testes in diabetic mice

**Figure-4:**
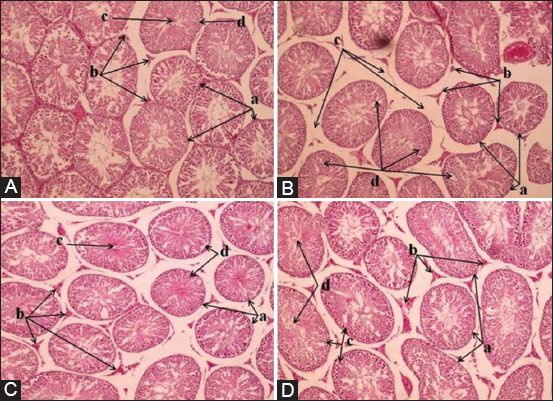
Bee venom enhances the histological architecture of the testis in diabetic mice, 10×. (A). a. Seminiferous tubules. b. Interstitial cells. c. Spermatid. d. Spermatogonia. (B). a. Seminiferous tubules. b. Interstitial cells. c. Interstitial vacuolization. d. Spermatogonia and spermatocyte vacuolization. (C) a. Seminiferous tubules. b. Interstitial cells. c. Spermatid. d. Spermatogonia. (D) a. Seminiferous tubules. b. Interstitial cells. c. Spermatogonia. d. Spermatid.

**Figure-5:**
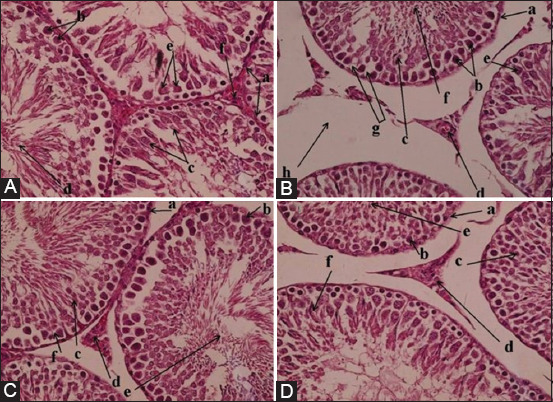
Bee venom enhances the histological architecture of the testis in diabetic mice, 40×. (A) a. Seminiferous tubules. b. Spermatogonia. c. Spermatocytes. d. Spermatid. e. Sertoli cells. f. Leydig cells. (B) a. Seminiferous tubule. b. Spermatogonia. c. Spermatocyte. d. Leydig cell. e. Sertoli cell. f. Spermatid. g. Spermatogonia vacuolization. h. Interstitial vacuolization. (C) a. Seminiferous tubule. b. Spermatogonia. c. Spermatocyte. d. Leydig cell. e. Spermatid. f. Sertoli cell. (D) a. Seminiferous tubule. b. Spermatogonia. c. Spermatocyte. d. Leydig cell. e. Spermatid. f. Sertoli cell.

The histological architecture of the testicular tissue was explored in mice of all groups after 35 days of treatment. The histological structure of the testes in the control mice showed typical architecture with the regular number of interstitial Leydig and Sertoli cells that lay between adjacent seminiferous tubules and inside them. Furthermore, the normal distribution of the Sertoli cells, spermatogonia, spermatocyte, and spermatid was seen clearly (Figures-4 and 5A).

Alloxan-induced mice showed various histological alterations of the testes, including reduced number and diameter of seminiferous tubules with shrunk and irregular shapes. Moreover, the interstitial space was increased with the reduced number of Leydig and Sertoli cells. Furthermore, vacuolization and mild sloughing of interstitial cells were seen between the seminiferous tubules. Similarly, vacuolization and initial degeneration of some spermatogonia and spermatocytes in seminiferous tubules were recognized (Figures-4 and 5B).

Administration of BV at two different concentrations caused gradual improvement of the impaired histological structure of the testes. The number, size, and shrinkage with the irregular shape of seminiferous tubules were returned to a nearly normal structure. Furthermore, the number of interstitial Leydig cells and seminiferous supported Sertoli cells was increased in response to BV. The wide interstitial space was reduced, in addition to the gradual reduction of spermatogonia and spermatocyte degeneration and vacuolization, as well as reduced interstitial cell sloughing and vacuolization (Figures-4, 5C, and D). The BV at 1 mg/kg appeared more potent and caused enhancement of altered histological structure of the testes.

## Discussion

In the past centuries, the incidence of diseases has dramatically increased due to simple medical intervention. Nowadays, the intervention and prevention strategies of diseases dissemination are developing in parallel with the development of the medical sector [25]. However, several diseases such as cancer and diabetes are still continuously prevalent affecting the global quality of life [7]. Despite global efforts, the prevalence is still rising, and any effective type of intervention is crucial in managing the global health issue [26].

Acupuncture, herbs, animal toxin, and others are part of alternative therapy that has been used for a long history in treating and preventing several health diseases in many territories, alongside standard medicine [27–29]. The T2DM is one of the leading public health concerns due to the absence of curative therapy and rising global incidence. Furthermore, nephropathy, retinopathy, cardiovascular, cancer, and neuropathy are the significant consequences of T2DM [30]. Several studies have focused on T2DM and its consequences through applying various complementary therapies [31–33].

Recently, animal toxins showed promising potential therapeutic alternative medication to prevent and manage several health issues [34, 35]. Apitoxin, BV, has various health benefits, including potential antidiabetic properties [36, 37]. Since male infertility is a consequence of T2DM, the antidiabetic effect of BV and subsequent assessment of restoring the fertility capacity and improving the histological structure of the testes are needed to study. Thus, this study is conducted to determine this relation and the regulatory role of BV in ameliorating infertility markers in male mice.

T2DM is recognized by the dysregulation of glucose and lipid utilization associated with impaired insulin production [38]. The T2DM is characterized by the increased level of circulatory glucose with abnormal insulin levels [39]. In this study, injected alloxan altered the concentration of glucose and insulin. This impairment was restored in response to BV in diabetic mice [23]. The BV decreased the blood glucose level with a high concentration of insulin that stimulated cellular glucose utilization [23]. The high level of insulin could be due to the ability of BV to alter the depolarization of the beta cell membrane, which facilitates the entering of calcium through its opening channels, stimulating insulin secretion [36, 37]. This result is promising for managing glucose and insulin levels in people with diabetes.

Disturbance of male fertility hormones leads to infertility and is a consequence of T2DM [15, 40]. The hormonal alteration includes a reduction in testosterone, FSH, and LH levels and an elevated level of prolactin [40, 41]. However, some studies showed a reduced level of testosterone and prolactin [42, 43] and an increased level of LH with no change in the FSH level [43]. The latter results are in line with this study’s results. The reduced level of testosterone and prolactin might be due to hyperglycemia that increased free radical production to induce oxidative stress, triggered testicular tissue, particularly Leydig cells, and reduced their function associated with reduced beta-cells function and increased insulin resistance [40, 43, 44]. In contrast, the decreased level of testosterone was the main reason for a high level of LH due to the feedback mechanism, whereas the high level of FSH might be due to dysfunction of Sertoli cells and seminiferous tubules that reduced inhibin secretion [45].

Furthermore, in this study, administration of BV ameliorated the effect of alloxan and caused elevation in the level of testosterone and prolactin, concomitant with a reduced level of FSH and LH. This result is unique as no study has been conducted to identify the role of BV using diabetic mice. The increased level of testosterone and prolactin could be due to the ability of BV to reduce the level of glucose and scavenging free radicals, restoring the proper Leydig and beta-cell function. This elevated testosterone level stimulated the pituitary gland’s feedback mechanism to reduce the FSH and LH levels. Furthermore, increased secretion of inhibin due to restoring the function of seminiferous tubules and Sertoli cells by BV might be the reason for FSH and LH levels. Accordingly, this result suggested an initial improvement of male fertility in diabetic conditions by BV.

Alteration of sperm quality is another consideration involved in developing infertility in diabetic conditions. Several studies identified remarkable deterioration of sperm characters in response to T2DM, including reduction of sperm viability and motility associated with elevated dead sperm and abnormal morphology, which consequently declined the quality of sperm [40, 43, 46]. These results are aligned with this study’s results. However, all deteriorations were reversibly restored to normal in response to BV. The high quantity of ROS produced in diabetic conditions directly interacts with testicular function, resulting in reduced quality of spermatozoa [40, 43]. This impairment was improved by increasing the activity of antioxidant enzymes concomitant with a reduced oxidation process in response to BV [47]. This could be the main reason to exert the low quality of sperm in natural and synthetic-induced T2DM. The previous study has determined the potential role of BV in ameliorating diabetic condition and their consequences. Only a single study assessed the adverse cytotoxic effect of BV on semen quality in healthy normal mice with a short duration of treatment [48]. In contrast, this study was conducted in alloxan-induced diabetic mice using two BV concentrations for 35 days. Therefore, the present study result is marvelous and unique, showing the distinct role of BV in enhancing sperm quality in diabetes conditions and subsequently improving infertility issues of diabetic patients.

Changes in the histological structure of the testes are observed in male fertility cases that are directly linked to T2DM. The histological alterations of the testes include marked reduction of Sertoli and Leydig cells with wide interstitial space, reduction in the diameter of the seminiferous tubules and changing of their shape, degeneration of spermatogonia and spermatocyte with seminiferous vocalization, and reduction the spermatid in the seminiferous tubules’ lumen [40, 43, 46, 49]. The results of these studies are in line with the present study result. Furthermore, BV showed an ameliorative effect on all testicular alterations through improving the cellular damages and enhanced testicular function alongside sperm quality and hormonal improvement. The T2DM-induced testicular deterioration could be due to the increasing level of ROS in response to hyperglycemia associated with an impaired antioxidant activity which promotes cellular damage resulting in a reduction in testicular functions [40, 43].

In contrast, BV might increase antioxidant enzyme activity and inhibit oxidation involved in scavenging the high level of ROS and improving the testicular alterations [47]. Furthermore, a study conducted by Regeai *et al*. [48] showed the toxic and destructive effect of BV on testicular tissue; however, the study used normal animals and short duration. The results of this study highlighted the potential therapeutic effect of BV in male infertility. However, further investigation is required to elucidate the exact role of BV in several diseases and disorders related to infertility.

## Conclusion

Assessment of the effect of BV on T2DM and the consequence of male infertility is crucial to identify the regulatory role of BV on glucose and insulin levels and improve male infertility. In this study, administration of BV showed glycemic control and enhanced fertility impairment in diabetic mice. Therefore, the BV could be a potential toxin to enhance infertility through antidiabetic action. However, further in-depth research is needed to quantify the mechanism of action of BV to ameliorate testicular deterioration and improve function in various conditions related to male fertility.

## Authors’ Contributions

SJJA and AME: Study design, practical works (induction of diabetes, BV treatment and animal monitoring, samples collection, and tests proceeding), and statistical analysis. TJH: Histology (prepared slides and histopathological reading). All authors have read and approved the final manuscript.
